# Examining the Correlation between Acute Behavioral Manifestations of Concussion and the Underlying Pathophysiology of Chronic Traumatic Encephalopathy: A Pilot Study

**DOI:** 10.13188/2332-3469.1000037

**Published:** 2018-05-11

**Authors:** M Byrd, CE Dixon, B Lucke-Wold

**Affiliations:** 1 Department of Exercise and Sports Psychology, West Virginia University, WV; 2 Department of Neurosurgery, University of Pittsburgh, PA; 3 Department of Neurosurgery, West Virginia University, WV

**Keywords:** Chronic traumatic encephalopathy, Concussion, Cognitive impairment, Alpha synuclein, Seroternergic staining, Impulsivity, Anger, Anxiety

## Abstract

Concussion in athletes can contribute to early neuropsychological changes that may be indicative of future neurodegenerative disease. One of the hallmark findings of chronic traumatic encephalopathy is anxiety and impulsive behavior that often develops early in the course of the disease. The behavioral dysfunction can be grouped into a broader category referred to as cognitive disruption. The current gold standard for diagnosing chronic neurodegeneration is post-mortem evaluation of tauopathy to identify neurofibrillary tau tangles in neurons. Few studies, however, have looked at clinical correlations between acute injury and chronic neurodegeneration in terms of behavior. This lack of focus towards translational study has limited advancements towards treatment. In this pilot investigation, the acute cognitive and emotional (anger, impulsivity, and anxiety) affects of concussion in a cohort of collegiate athletes (n = 30) are examined and compared to findings in the post-mortem pathologic features of chronic traumatic encephalopathy. Specifically, the role of the seroternergic system with alpha synuclein and tauopathy staining and the potential for early clinically relevant behavioral and pharmaceutical interventions was investigated. The purpose was to determine if athletes began demonstrating cognitive disruption present in post-mortem evaluation during the acute phase of injury. The acute data was collected via questionnaires within ten days of the athletes’ concussion diagnosis. Results demonstrated that 11 of 30 athletes (36%) scored in a diagnosable range of anxiety post-concussion, and athletes scored above the norm in state-anger (*M* = 22.9, SD = 9.99), indicating severe emotional disturbance. A limitation is that due to the long time frame from acute injury to the development of neurodegeneration individual athletes cannot be tracked in longevity thus limiting the findings to the realm of correlation. The findings from this pilot study warrant further investigation into the neuropsychological aspects for how to manage concussion and prevent degenerative disease.

## Introduction

As reported by the 2012 Zurich consensus statement on concussion in sport and in an evidence-based systematic review [1,2], athletes with a history of previous concussion are at a higher risk of sustaining subsequent concussions. Thus, prior concussions actually predispose an athlete to obtaining future concussions. Furthermore, the rehabilitation process following a first concussion verse a subsequent concussion is quite different because recovery times may not be uniform. Additionally, multiple concussions have been shown to contribute to neurodegenerative disease such as chronic traumatic encephalopathy [3]. Concussions and the development of chronic neurodegeneration may share similar psychological and behavioral responses however research into this important topic is currently limited.

Sport concussion management and diagnosis is considered one of the most complex facets of sport medicine as not all concussions “look” the same and not all concussions lead to neurodegeneration [4]. Brain injuries when the athlete is rendered unconscious, amnestic, and disoriented are easier to detect, but more than 90% of sport-related head injuries result in no observable Loss of Consciousness (LOC) or amnesia and only slight disorientation with subtle symptoms such as headaches and poor concentration [5,6]. Furthermore, subconcussive impacts have still been shown to contribute to neurodegeneration but are not readily detected clinically [7].

A recent study looked at the acute cognitive effects following concussion in a three-year prospective cohort study with 1631 football players from 15 colleges [8]. Ninety-four football players who had a concussion were included in the study and were matched to a non-injured control from each injured player’s team. The results demonstrated that the athletes exhibited the most severe cognitive impairments at the time of injury and through preinjury day 2. Symptoms resolved within seven days after concussion for 91% of players with concussion, with roughly 10% of athletes requiring more than one week to fully resolve symptoms. No studies however have mapped initial concussion symptoms to symptoms that develop with chronic neurodegeneration, especially for those 10% with persistent symptoms.

Athletes who experience acute and prolonged cognitive symptoms likely develop frustration in response to symptoms and additional distress that may lead to anxiety, impulsivity, and avoidance of social situations [9]. Interestingly, these same symptoms are often the first reported symptoms in patients eventually diagnosed with chronic traumatic encephalopathy [10]. Collegiate athletes may appear withdrawn in classes or avoid situations with teammates and friends that may feel anxiety inducing after sustaining a concussion. Feelings of anxiety may lead to development of depression, which may lead to further cognitive compromise and additional emotional distress. Over the course of years these symptoms may continue to persist and intensify switching from subacute to chronic disease. The inter-play between cognitive and emotional symptoms can be more debilitating than the actual injury itself and so a better understanding of both can lead to better treatment and rehabilitation outcomes.

In addition to somatic and cognitive symptoms, athletes often have emotional and psychological symptoms following concussion. Psychological and emotional symptoms could stem from both brain tissue damage, as well as from psychosocial sequelae [9]. The seriousness of concussions has previously been downplayed and ignored in professional and collegiate sport, but due to forced retirement of high-profile athletes who have sustained multiple concussions, the gravity of the effects of sports concussions is finally being acknowledged [11]. This attention has led to more research into the epidemiology and etiology of concussions, but there is still a lack of research pertaining to the emotional symptoms following sport concussion. In this pilot paper, we investigate acute symptoms in collegiate athletes and compare these findings to neuropathologic features of disruption of the seroternergic system evidence by tau and alpha synuclein staining in deceased professional athlete brains diagnosed with chronic traumatic encephalopathy postmortem. The purpose of this study was two-fold. The first objective was to determine if collegiate athletes demonstrated emotional disturbance within 10 days of concussion diagnosis. The second objective was to compare the findings from the collegiate athletes to the role of the seroternergic system with alpha synuclein and taupothy staining and the potential for early clinically relevant behavioral and pharmaceutical interventions. As this was a pilot study, few hypotheses were generated. For the acute phase, it was hypothesized that athletes would exhibit elevated scores of anxiety, anger, and impulsivity as compared to normative data of college-aged students. For the pathology phase, it was predicted that serotenergic alpha synuclein would be increased potentially contributing to the underlying behavioral symptoms.

## Methods

### Participants

Participants were 30 collegiate male (n = 23) and female (n = 7) athletes from soccer (n = 3), volleyball (n = 2), football (n = 22), lacrosse (n = 1), diving (n = 1), and basketball (n = 1) who were diagnosed with a sport-related concussion during the Fall 2015 through Fall 2016 athletic seasons. Seven athletes were excluded from the final data set for incomplete data or not completing surveys within 10 days of concussion. Athletes ranged in year in school from freshman to graduate student and had a mean age of 21.47 (*SD* ± 2.01) years old. Six athletes were diagnosed with more than one concussion in the same year, with a mean of 1.67 (*SD* = .96) concussions in their collegiate career and 16 of 30 athletes reported self or others noticing a difference in mood or behavior since their concussion, and one athlete reported having previously been diagnosed with a mental illness (anxiety disorder).

### Instrumentation

Athletes were given the following quantitative assessments in order to assess acute emotional symptoms of anxiety, anger, and impulse within 10 days of their diagnosed concussion (1) Demographic questionnaire assessing athletic sport history, age, sport type, concussion history, the observed mechanism of the concussion (i.e., acceleration or rotational forces applied to head), and mental health history. (2) Post-Concussion Symptom Scale. Concussion symptoms were measured using the post-concussion symptom scale (PCSS; Lovell & Collins, 1998) of the Immediate Post-Concussion Assessment and Cognitive Test (ImPACT Version 2.0, Lovell, Collins, Podell, Powell, & Maroon, 2000). It is a 21-symptom checklist to document and track concussion symptoms on a 7-point Likert scale (0 = no experience of a symptom to 6= severe symptom). (3) Barratt Impulsiveness Scale-Version II. The Barratt Impulsiveness Scale-II (BIS-II; Patton, Stanford, & Barratt, 1995) has 30 questions that are ranked on a 4-point Likert scale (1 = rarely/never to 4 = almost always/always), with 4 indicating the most impulsive response. The higher the summed score for all items, the higher level of impulsiveness. (4) State-Trait Anger Inventory-2. The State Anger subscale of the State-Trait Anger Inventory-2 was used to measure state anger (STAXI-2; Spielberger, 1999). It is comprised of 15-items measured on a 4-point Likert type scale (1 = not at all/almost never, 4 = very much so/almost always). Generalized Anxiety Disorder 7-item. The Generalized Anxiety Disorder 7-item screening measure (GAD-7; Spitzer, Kroenke, Williams, & Lowe, 2006) has seven items answered on a four point Likert Scale (0 = not at all sure; 3 = nearly every day). The GAD-7 is a clinical anxiety assessment, with a score above 10 indicating a probable diagnosis of anxiety, and a score above 15 indicating severe anxiety.

### Procedure

Institutional review board was obtained from two institutions for the study. Participants were recruited via team doctors and athletic trainers. Questionnaires were distributed to participants via in person or email using Qualtrics online software. Participants completed the questionnaires within 10 days of their concussion diagnosis.

#### Post-mortem CTE Tissue Preparation

Chronic traumatic encephalopathy brain tissue from three deceased professional athletes was paraffin embedded, sliced, and stained with standard immunohistochemical procedures including xylene and alcohol washes, antigen retrieval, quenching, permeabilization, and primary and secondary antibody staining. Primary antibodies included paired helical filament and alpha-synuclein in the dorsal raphe nucleus (the primary serotenergic brain region) with DAB staining. Two brain samples were stained with PHF and the remaining one with alpha-synuclein.

## Results

Descriptive statistics were conducted on all dependent variables. On the STAXI-2, athletes scored a mean of 22.9 (*SD* = 9.99). As comparison, normative for males on this inventory is a mean of 19.47 (*SD* = 7.14) and for females is 18.17 (*SD* = 5.50) (Spielberger, 1988). On the BIS-II, athletes scored a mean of 66.53 (*SD* = 10.77). A BIS-II score one standard deviation above the mean is used to designate high impulsiveness (Patton Stanford, & Barratt, 1995). 10 athletes scored at least one standard deviation about the norm mean 1 to 10 days post-concussion. Athletes scored a mean of 7.4 (*SD* = 5.6) on the GAD-7. Eleven of 30 athletes scored above a 10 on the GAD-7, with three of those athletes scoring above 15, indicating criteria for the diagnosis of an anxiety disorder. In regards to concussion symptoms, headache was the most frequently with the most intensity among the participants (*M* = 3.27, *SD* = 1.84), followed by difficulty remembering (*M* = 2.87, *SD* = 2.24), and irritability (*M* = 2.57, *SD* = 2.19).

Linear regression was calculated to determine if athletes’ scores on the anxiety, anger, or impulsivity measures were predicted by their reported symptoms on the PCSS. A significant regression equation was found on the anxiety measure (F(1, 29) = 20.18, *p* <.001) with an adjusted R^2^ of .398. Table 1 for regression coefficients. Regression analysis from the impulsivity and anger measure were not significant.

**Figure 1:**
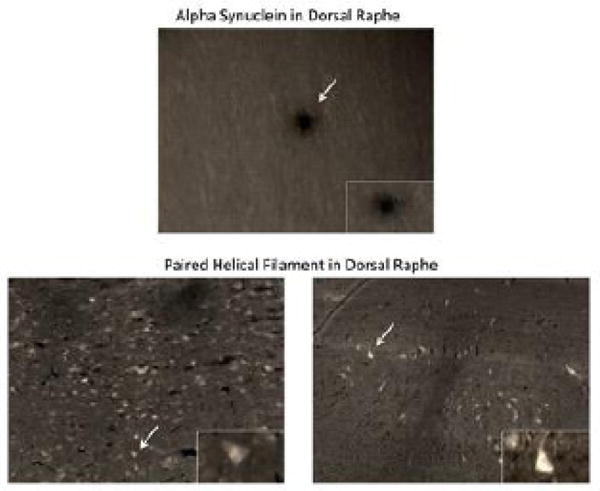
Alpha-synuclein and paired helical filament staining in the dorsal raphe nucleus. Damage to the dorsal raphe nucleus has been closely linked to mood disorders due to the widespread projections of the serotenergic system. Concussion likely causes early protein aggregation within the dorsal raphe nucleus leading to behavioral dysfunction and the progression towards neurodegenerative disease.

The first chronic traumatic encephalopathy brain was from a 41 year old retired football player who had a history of anxiety, depression, and agitation in the years prior to his death. The second brain sample was from a 40 year old retired wrestler who had a history of memory loss, depression, and drug abuse prior to his death. The third brain sample was from a 36 year old retired football player who had neuropsychiatric symptoms and depression prior to his death. We found that PHF and alpha-synuclein were both significantly elevated in the dorsal raphe nucleus indicating a pathologic correlate for persistent behavioral changes (Figure 1).

## Discussion

Chronic traumatic encephalopathy is currently only diagnosed by pathologic evaluation. Because of this limitation, it is not known how many individuals are susceptible to developing the disease after concussion. One of the primary barriers is the time gap between last concussion and onset of symptoms of chronic traumatic encephalopathy. What has been poorly investigated however is the type of symptoms following concussion that may be a predisposing indication for the likelihood of developing neurodegeneration. One of the key circuitry systems within the brain that is likely damaged with concussion is the dorsal raphe nucleus [12]. The dorsal raphe nucleus is the control center of the serotenergic fiber tracts within the brainstem. The fiber tracts are susceptible to injury and shearing during the impact of a concussion due to their broad reaching projections [13]. Not surprisingly, this important system has been closely linked with anxiety and impulsivity [14].

In this pioneering pilot study, we sought to provide a new way to look at symptoms post-concussion as a potential future predictor of the development of neurodegeneration. What we found was that 11/30 athletes post-concussion scored above a 10 on the GAD-7, indicating that they met criteria for an anxiety disorder, and 3/30 athletes scored above a 15, indicating severe anxiety. Anxiety scores were also significantly correlated with symptoms on the PCSS. In a recent prospective study of 341 adults with mild traumatic brain injury (TBI), over 45% of individuals had persistent concussion related mood disorders at 1 year [15]. These persistent symptoms may be indolent markers of a more insidious underlying pathophysiology. Our group recently showed that acute cellular injury pathways such as endoplasmic reticulum stress and oxidative stress contribute to pathologic protein aggregates within the brain following early injury [16,17].

Axonal shearing within the serotenergic system can activate these pathways and lead to progressive injury and degeneration. We report in this paper significant positive staining for both tauopathy and alpha-synuclein within the dorsal raphe nucleus from CTE specimens. The deceased athletes displayed very similar symptoms to the collegiate athletes highlighted above, but the symptoms persisted for years. This underlying pathophysiology likely correlated with acute anxiety behavior following the initial concussions as well as the resurgence of mood-based symptoms with the onset of chronic traumatic encephalopathy. Of note, there is significant overlap of symptoms for post-concussion syndrome and those of initial chronic traumatic encephalopathy. This is due to the shared underlying cellular mechanisms at play during both acute injury and chronic injury that leads to degeneration [18]. Due to the long duration between initial injury and the development of chronic traumatic encephalopathy, one of the limitations of the study was that we had to look at acute behavioral changes post-concussion in collegiate athletes and then correlate this to pathology seen in deceased professional athletes. Ideally, the collegiate athletes could be tracked over years, but currently no prospective studies have been conducted.

The key point of this pilot study is to emphasize research into new diagnostic criteria for defining chronic traumatic encephalopathy. Pathologic changes are occurring within the brain much earlier than the initial onset of symptoms and post-mortem diagnostic evaluation. In order to prevent disease progression and provide treatment to restore the damaged serotenergic system, it will be necessary to diagnose the disease early. We propose that symptom manifestation can be a useful tool towards this mean. Early identification of individuals with persistent anxiety following concussion can group them into a cohort that may be more susceptible for developing chronic traumatic encephalopathy. Further, return to play protocols and concussion treatment should include an anxiety specific measure to better detect anxiety symptoms and behavioral manifestations. In these individuals, more extensive cognitive behavioral therapy and pharmaceutical approaches should be employed than for the cohort without persistent mood disorders. Going forward it will be necessary to prospectively map symptoms to microstructural findings on advanced imaging modalities in order to provide the best early diagnostic protocol. This pilot study also indicates the need for better long-term studies to determine the true incidence of CTE amongst former competitive athletes.

## Figures and Tables

**Table 1:** Regression Coefficients Predicting Athletes’ Anxiety.

Model	Predictor Variables	*B*	*SE*	β	*t*	*p*	Adjusted R2
1	PCSS	0.12	0.027	0.647	4.492	0.001***	0.398

Unstandardized regression coefficient, SE, standard error, β, standardized regression coefficient, *t*, obtained t value, *p* probaibility, R2, proprotion vairance explained.

*** p < .001
